# BET Inhibition Suppresses S100A8 and S100A9 Expression in Acute Myeloid Leukemia Cells and Synergises with Daunorubicin in Causing Cell Death

**DOI:** 10.1155/2018/5742954

**Published:** 2018-05-31

**Authors:** Helen J. S. Stewart, Sabah Chaudry, Asante Crichlow, Freya Luiling Feilding, Timothy J. T. Chevassut

**Affiliations:** Research Building, Brighton and Sussex Medical School, University of Sussex, Brighton BN1 9PS, UK

## Abstract

S100A8 and S100A9 are both members of the S100 family and have been shown to play roles in myeloid differentiation, autophagy, apoptosis, and chemotherapy resistance. In this study we demonstrate that the BET-bromodomain inhibitor JQ1 causes rapid suppression of* S100A8* and* S100A9* mRNA and protein in a reversible manner. In addition, we show that JQ1 synergises with daunorubicin in causing AML cell death. Daunorubicin alone causes a dose- and time-dependent increase in S100A8 and S100A9 protein levels in AML cell lines which is overcome by cotreatment with JQ1. This suggests that JQ1 synergises with daunorubicin in causing apoptosis via suppression of S100A8 and S100A9 levels.

## 1. Introduction

Acute myeloid leukemia is the most common leukemia in adults accounting for around 80% of cases [1]. It is a clonal disorder of the myeloid lineage of white blood cells characterised by the arrest of differentiation of immature ‘blast' cells in the bone marrow. Diagnosis and posttreatment monitoring of AML are generally accomplished using a combination of karyotype analysis and reverse transcriptase polymerase chain reaction (RT-PCR) or fluorescent in situ hybridization (FISH) for specific abnormalities. However around half of AML patients have a normal karyotype and intermediate prognosis. Furthermore, with limited exceptions little improvement in outcome for AML patients has been achieved with only around 35% of younger fit patients cured of their disease. Hence, additional cellular targets need to be identified in order to develop new chemotherapeutic treatment strategies.

S100A8 and S100A9 are part of the 22 member calcium binding EF-hand containing S100 superfamily of proteins that function predominantly in the innate immune system [2]. These low molecular weight proteins exhibit both cell type and tissue specific expression. S100A8 and S100A9 are able to form homodimers, heterodimer, and oligomers which can have distinct and even antagonistic functions. Both proteins are particularly highly expressed in cells of the myeloid lineage, particularly neutrophils where they account for around 45% of total cytoplasmic protein. Recent studies have highlighted S100A8 and S100A9 as having an important role in cancer pathogenesis. High levels of both proteins are found in cancer tissues with increased expression appearing to be a marker of tumour aggressiveness [3]. In AML, high levels of S100A8 [4] and S100A9 are correlated with poor overall survival predominantly in patients with myelomonocytic differentiation (M4, M5) [4, 5] and in* de novo* pediatric AML patients [4], whilst low S100A8 and S100A9 levels correlate with good prognosis in childhood AML with IDH1/2 mutations [6]. Furthermore, high expression of S100A8 is associated with chemoresistance in AML [7] whilst calprotectin (S100A8/S100A9 heterodimer) causes glucocorticoid resistance in infant ALL [8]. S100A8 induced chemoresistance is thought to be caused by promoting autophagy and antiapoptotic effects [7]. These studies suggest that targeting S100A8 and S100A9 may lead to improved outcomes not only in treatment of AML but also for many solid tumours. Therapies might have antimetastatic effects and help overcome drug resistance.

Targeted inhibition of BRD4, a member of the BET-bromodomain family of transcriptional regulators, has been demonstrated to have therapeutic potential in AML [9]. BET-bromodomain proteins act by ‘reading' histone acetylation marks, thus facilitating transcriptional activation. Antileukemic mechanisms induced by BET inhibitors, such as JQ1, are currently not well understood but are believed to involve suppression of MYC.

In this study, we show that in AML cells S100A8 and S100A9 expression is downregulated by JQ1 and upregulated by the anthracycline daunorubicin. Furthermore we show that JQ1 synergises with daunorubicin to cause apoptosis in AML cell lines. We hypothesize that JQ1 stimulates apoptosis by overcoming autophagy in an S100A8/A9-dependent manner.

## 2. Methods

### 2.1. Materials

Penicillin, streptomycin, L-glutamine, daunorubicin hydrochloride, Histopaque 1077, and RPMI 1640 were from Sigma (Poole, UK). TRIzol and reverse transcription polymerase chain reaction (RT-PCR) primers were purchased from Invitrogen (Paisley, UK); foetal calf serum was from Biosera (Ringmer, UK) and WST-1 from Roche (Welwyn Garden City, UK) 4-20% gradient gels from (Bio-Rad). The QuantiTect reverse transcription kit was from Qiagen (Manchester, UK) and TaqMan probes were from Life Technologies. Antibodies against S100A8 (ab180735) and S100A9 (ab63818) were from Abcam (Cambridge, UK). Anti-cleaved caspase 3 Asp 175 (#9661) was from Cell Signalling Technology (Hitchin, UK); anti-rabbit Alexa Fluor 488 from Life Technologies (Paisley, UK); and anti S100A8/S100A9 heterodimer from R&D systems (Abingdon, UK)

### 2.2. Microarray Analysis

OCI-AML3, KG1, and THP-1 cells were treated with 0.5 *μ*M (+)-JQ1 for 16 hours in triplicate experimental biological replicates. RNA was extracted with the TRIzol reagent and analysed using an Agilent 2100 bioanalyzer. All RNA used had a RNA integrity number (RIN) value of greater than 8.0. Transcriptional profiling for control and treated cells was performed using the Illumina expression microarrays. Raw data was normalized with a variance stabilizing transformation followed by robust spline normalization using the R package Lumi. Expression changes of genes were calculated by comparing the expression levels of treatment versus control untreated.

### 2.3. Leukemia Cell Lines and Patient Samples

Human Leukemia OCI-AML3 (FAB M4) and OCI-AML2 (FAB M4) and were kind gifts from Dr Terry Gaymes (Kings College, London, UK) KG1 (FGFR1OP2-FGFR1 rearrangement), and THP-1 (MLL-AF9) cells were a kind gift from Dr. Lisa Woodbine (Genome Centre, Sussex University). Cells were cultured in RPMI 1640 supplemented with 10% foetal calf serum, 2mM-glutamine, penicillin (100 IU/mL), and streptomycin (100 *μ*g/mL). The identity of the OCI-AML3 cell line was confirmed by carrying out a restriction-sensitive PCR assay for mutated DNMT3A at codon R882 that this cell line harbours [10].

A total of 18 AML patient samples were studied (Table 1). All primary bone marrow aspirates were taken from routine diagnostic specimens after informed consent of the patients. The project received approval from the local ethics committee (*The Brighton Blood Disorder Study, references 09/025/CHE and 09/H1107/1*) and was conducted in accordance with the Declaration of Helsinki. Mononuclear cells were isolated by Histopaque 1077 density gradient purification. Peripheral blood was taken from healthy volunteers. Monocytes and lymphocytes were prepared from peripheral blood mononuclear cells by differential adhesion [11, 12]

### 2.4. Cell Viability Assays

For cell lines, cell viability was assessed using a WST-1(4-[3-(4-iodophenyl)-2-(4-nitrophenyl)-2H-5-tetrazolio]-1,3-benzene disulfonate) assay. 20,000 cells were plated per well in 96-well plates and were incubated in the presence of compound for the times indicated. Two to four hours after the addition of WST-1, plates were read at 450 nm in a Biotek Synergy HT plate reader (Biotek, Potton, UK) and Gen5 version 2.04 software. The viability of untreated cells was set as 100%, and viability in other groups was calculated by comparing the optical density readings with the control. All compounds were dissolved in dimethylsulfoxide and stored at -20°C. Dilutions in PBS were used for experiments [12].

### 2.5. Immunolabelling

Cells were smeared onto poly-l-lysine coated slides and air dried for 1 hr. Preparations were fixed in methanol -20°C for 5 min and then blocked in antibody diluting solution containing 0.1% Tween 20 for 30 min. Rabbit anti-s100A8 (1:200) s100A9 (1:200) or S100A8/S100A9 heterodimer (1:250) were applied overnight at 4°C. After washing through PBS, cells were incubated in anti-rabbit Alexa Fluor 488 (1:250) for 30 min. After a final wash through PBS, the slides were incubated in DAPI (4′,6-diamidino-2-phenylindole) for 5 min and then mounted in Citifluor anti-fade mounting medium and viewed for fluorescence using a Leica DM 5000B microscope fitted with a Leica DPC300FX digital camera (Leica, London, UK).

### 2.6. Quantitative RT-PCR

Total RNA was extracted from cells using TRIzol reagent according to the manufacturer's instructions. RNA(1 *μ*g) was reverse transcribed using a QuantiTect cDNA kit and the PCR reactions were performed using the TaqMan Gene Expression Assays (Applied Biosystems)* c-MYC* (Hs00811069_g1),* S100A8* (Hs00374264_g1),* S100A9* (Hs00374264_g1), and* GAPDH* (Primer design) using standard TaqMan reagents and protocols on a MX3500 (Stratagene) qRT-PCR machine. The ΔΔct method was used for relative expression quantification using the average cycle threshold for GAPDH RNA to normalize gene expression levels between samples. All experiments were performed in triplicate and expression levels were compared between JQ1 and (-)-JQ1-treated cells using a two-tailed* t*-test with P values < 0.05 [13].

### 2.7. Western Immunoblotting

OCI-AML3 cells were treated with 0.5 or 1 *μ*M JQ1 for 72 hr and then harvested. Proteins were extracted in sample buffer containing protease inhibitor tablets. Samples were then diluted in loading buffer (with *β*-mercaptoethanol), boiled for 7 min, and subjected to gel electrophoresis on 4-20% SDS polyacrylamide gels. A total of 50 *μ*g of cell extract was loaded per lane. Proteins were electrophoretically transferred onto a PVDF membrane for 1 hr at 30mA constant current. Membranes were blocked with 5% milk for 1 hr at room temperature and then rotated overnight at 4°C with primary antibody S100A8 (1:500), S100A9 (1:500), and GAPDH (1:10000) in TBS 0.1% Tween. Fluorescently linked secondary antibodies were used at a dilution of 1:20,000 for 1 hr. Blots were analysed using a Licor Imaging system.

## 3. Results

### 3.1. Microarray

#### 3.1.1. *S100A8* and* S100A9* mRNA Are Expressed by Leukemia Cell Lines and Patient Samples

**Figure 1:**
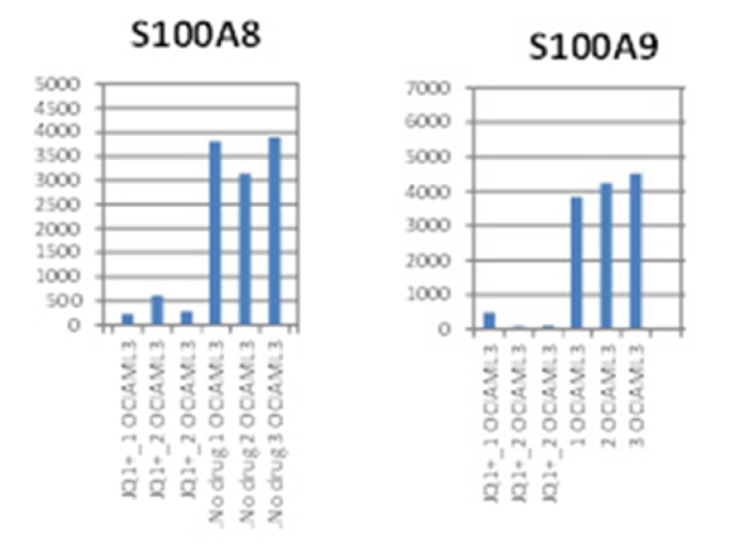
Summary of S100A8 and S100A9 microarray analysis. OCI-AML3 cell lines were treated with 0.5*μ*M JQ1 for 16hr.

**Figure 2:**
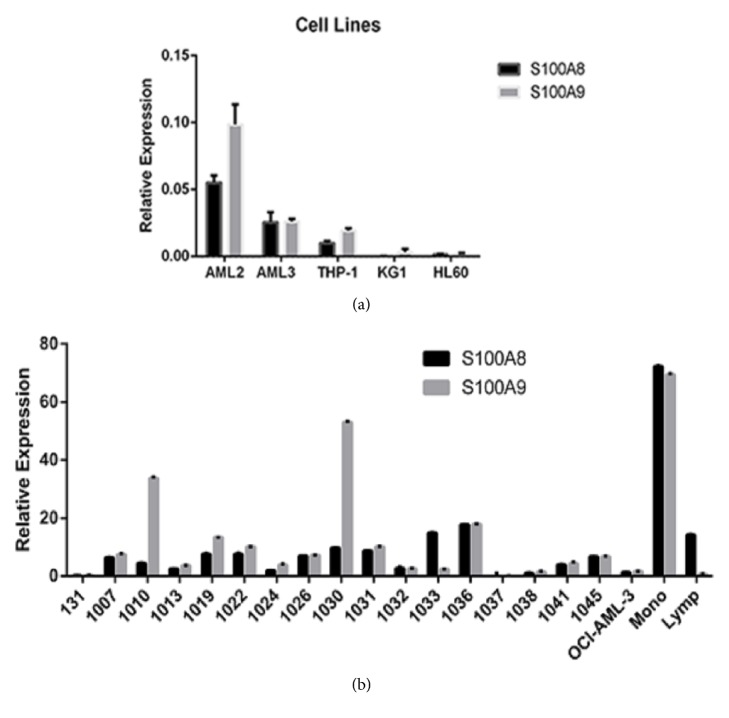
***Expression of S100A8 and S100A9 mRNA in AML cell lines***. qRT-PCR was used to analyse the expression of S100A8 and S100A9 mRNA in cell lines (a) and mononuclear cells from patient and healthy donors (b). Results were calculated using the ΔΔct method and expressed relative to the housekeeping gene GAPDH. Note that in patient samples the levels of both* S100A8* and* S100A9* mRNA are exceedingly high compared to GAPDH.

Microarray experiments conducted on leukemia cell lines treated with the bromodomain inhibitor JQ1 showed that JQ1 causes a significant downregulation of* S100A8* and* S100A9* transcripts in OCI-AML3 cells (Figure 1). Downregulation was also seen to a lesser extent in THP-1 cells; however, these cells had lower basal levels of both S100 genes. KG1 cells expressed extremely low levels of* S100A8* or* S100A9* mRNA (not shown). Thus, we initially conducted experiments to confirm the levels of* S100A8* and* S100A9* mRNA in these cell lines and to determine* S100A8* and* S100A9* expression in bone marrow mononuclear cells (BMMCs) and peripheral blood mononuclear cells (PBMCs). Results from qPCR experiments on cell lines (Figure 2(a)) revealed levels of transcripts varied across the cell lines with HL60 and KG1 cells expressing very little of the mRNAs confirming our microarray data and previous publications [1]. Likewise, there was considerable variation in mRNA expression across the BMMCs from 18 AML patients (Figure 2(b)). However, levels were all significantly higher than in OCI-AML3 cells. Patient characteristics are shown in Table 1. Monocytes prepared from healthy donors showed very high levels of both S100 transcripts as expected serving as a positive control (see Figure 2(b)).

#### 3.1.2. BET-Bromodomain Inhibitor JQ1 Downregulates S100A8 and S100A9 in AML Cells and Primary Blood Cells

**Figure 3:**
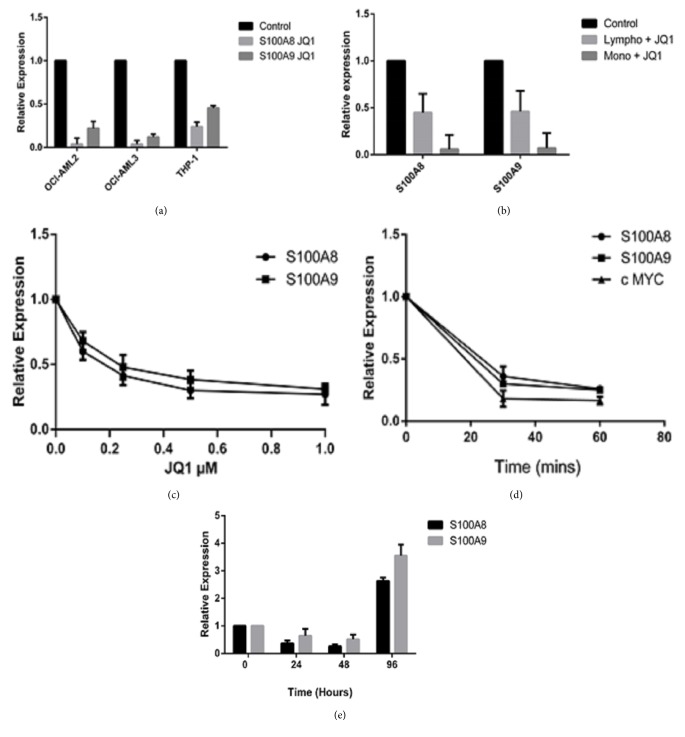
***Regulation of S100A8 and S100A9 mRNA expression by BET-bromodomain inhibition***. qRT-PCR was used to show that regulation of* S100A8* and* S100A9* mRNA by JQ1 in AML cell lines (a) and primary mononuclear cells (b). OCI-AML3 cells were used to show the effect of JQ1 was (c) dose-dependent, (d) time-dependent, and (e) reversible. 0.5*μ*M JQ1 was used for time course and reversibility experiments. Results are expressed relative to GAPDH (mean ± SEM, n=3).

**Figure 4:**
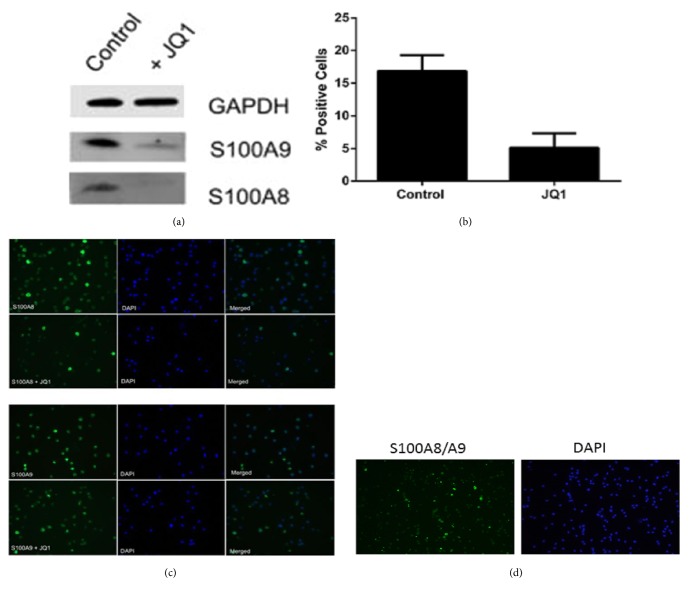
**Effect of JQ1 on S100A8 and S100A9 protein expression in the OCI-AML-3 cell line**. Cells were treated with 0.5*μ*M for 48 hr; then extracts were subject to Western blotting (a) or immunolabeling for S100A8 and S100A9 (b). Counts of highly S100A8 positive cells are shown in (c) (mean ± SEM, n=3). Immunolabeling for S100A8/S100A8 heterodimer is shown in panel (d).

We next sought to confirm the effects of JQ1 on* S100A8* and* S100A9* mRNA. Both cell lines and monocytes and lymphocytes from healthy volunteers responded to JQ1 with a robust decrease in both* S100A8* and* S100A9* mRNA (Figures 3(a) and 3(b)). qPCR confirmed that JQ1 suppresses* S100A8* and* S100A9* mRNA in a dose- and time-dependent manner (Figures 3(c) and 3(d)). S100A8 mRNA was marginally more suppressed than S100A9 mRNA after JQ1 treatment in all cell lines tested. Using OCI-AML3 cells we found that maximal suppression occurred within 30 mins of treatment with 0.5*μ*M JQ1 and the time frame was comparable to* c-Myc* downregulation (Figure 3(d)). Suppression of these mRNAs was reversible with* S100A8* and* S100A9* exceeding control levels within 24hr of removal of JQ1 (Figure 3(e)). Western blotting showed that JQ1 treatment caused significant downregulation of S100A8 protein with S100A9 downregulating to a much lesser extent (Figure 4(a)). This was confirmed by immunolabelling (Figure 4(b)) which revealed that S100A8 was not uniformly expressed in the cells. Approximately 16% of cells were very brightly S100A8 positive in control experiments reducing to 5% in cells treated with JQ1 0.5*μ*M for 72 hr (Figure 4(c)). S100A9 immunolabelling was much more homogenous though after JQ1 treatment the appearance of the labelling was more punctate (not shown). Immunolabelling with an antibody that detects S100A8/A9 (calprotectin) only when they are associated as heterodimers revealed that majority of S100A8 and S100A9 are not in heterodimeric form with less than 2% of cells expressing the S100A8/A9 heterodimer (Figure 4(d)).

### 3.2. The Effect of JQ1 on Patient Samples

**Figure 5:**
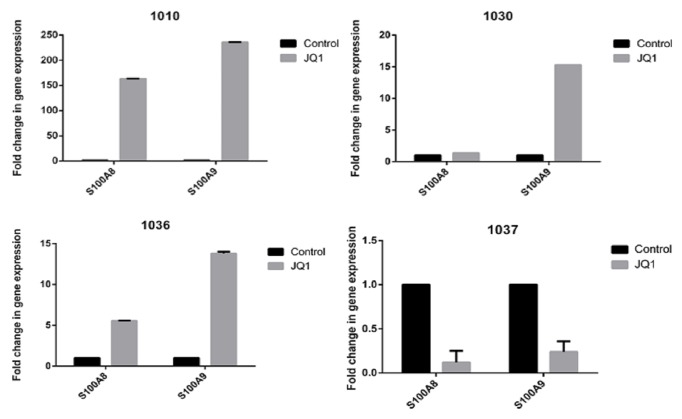
**Effect of BET-bromodomain inhibition on AML patient samples**. BMMCs from four patients were treated with 0.5*μ*M JQ1 for 48hr and then* S100A8* and* S100A9* mRNA levels quantified by qRT-PCR.

We tested 0.5*μ*M JQ1 of* S100A8* and* S100A9* mRNA on BMMC's isolated from patient samples. Unlike cell lines and cells from healthy volunteers, JQ1 often caused an increase in* S100A8* and/or* S100A9* mRNA expression (Figures 5(a)–5(c)). In some patient samples however JQ1 suppressed expression of either or both mRNAs (Figure 5(d)).

### 3.3. JQ1 Synergises with Daunorubicin in Inducing AML Cell Death

**Figure 6:**
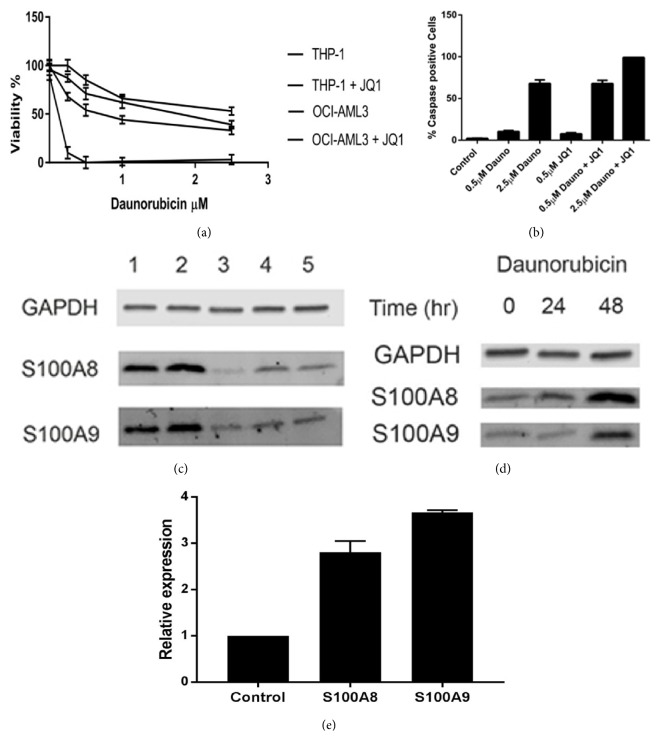
**Effects of JQ1 and daunorubicin on S100A8 and S100A9 expression in OCI-AML3 cells**. Cells were incubated in medium containing various concentrations of daunorubicin with or without 0.5*μ*M JQ1 for 48 hr at 37°C. After incubation cell viability (a) was measured using WST-1 reagent and detection at 450nm or activated caspase 3 was detected by immunolabelling and caspase 3 positive cells counted (mean ± SEM, n=3). In (a) the legend corresponds to the data lines from top to bottom (b). Western blotting of (c) OCI-AML3 cells treated with lane 1: daunorubicin 0.5*μ*M; 2: daunorubicin 2.5*μ*M; 3: JQ1 0.5*μ*M; 4: daunorubicin 0.5*μ*M plus JQ1 0.5*μ*M; 5: control for 48hr, and (d) time course of induction of S100A8 and S100A9 protein expression by 0.5*μ*M daunorubicin. (e) shows changes in mRNA expression after 24 hr treatment with 0.5*μ*M daunorubicin.

As we have shown that JQ1 suppresses S100A8 and S100A9 expression and S100A8 has been shown to promote chemoresistance in leukemia cells [7], we hypothesised that JQ1 may enhance the effects of drug action in AML cells. Cell viability assays showed that JQ1 treatment synergised with the anthracycline daunorubicin in causing cell death in OCI-AML 3 cells (Figure 6(a)). This was also seen in THP-1 cells (Figure 6(a)). Furthermore, immunolabelling for activated caspase 3 D175 reveals a synergistic increase in apoptosis when both drugs are used together (Figure 6(b)). Western blotting experiments showed that daunorubicin causes a dose- and time-dependent increase in S100A8 and S100A9 protein expression that was overcome by 0.5*μ*M JQ1 (Figures 6(c) and 6(d)). An increase was also seen in mRNA levels (not shown).

## 4. Discussion

In this study we have shown that the BET-bromodomain inhibitor JQ1 suppresses expression of* S100A8* and* S100A9* mRNA and protein in AML cell lines and PBMCs. This effect is dose-dependent and reversible.* S100A8* and* S100A9* downregulation occurs rapidly with a time course paralleling* c-myc* downregulation, a well-characterised target of BET-bromodomain inhibitors. Application of JQ1 to primary AML cells however does not always result in* S100A8* and S*100A9* downregulation. Rather, in most, but not all cases, upregulation of mRNA is seen. This may be because the effects of JQ1 are influenced by the mutational status of the leukemia. This observation needs to be explored more thoroughly in a larger cohort of patient samples. Immunolabelling experiments suggest that S100A8 and S100A9 are largely present in AML cells as homodimers or oligomers as an antibody specifically detecting S100A8/S100A9 heterodimer detected very few positive cells.

Furthermore, we show that JQ1 synergises with the anthracycline daunorubicin in causing AML cell death and may play a role in overcoming chemoresistance. We hypothesize that JQ1 helps overcome chemoresistance by reducing S100A8 and S100A9 levels in AML cells which in turn leads to inhibition of autophagy. Our results support and complement data of Yang et al., 2014, who show that overexpression of S100A8 leads to chemoresistance in some leukemia cell lines, one of which (HL60) is an AML cell line. This is caused by S100A8 induction of autophagy via binding to autophagy regulator BECLIN1 [7]. In addition, elevated S100A8 and S100A9 cause glucocorticoid resistance in pediatric acute lymphoblastic leukemia [8]. It has recently been reported that JQ1 inhibits autophagy in an AML cell line bearing an NPM1 mutation (OCI-AML3 cell line used here) or an MLL fusion by a mechanism involving NPM1 and HEXIM1 [14]. Furthermore additional support for JQ1 playing a role in autophagy comes from the observation that, in addition to suppression of S100A8 and S100A9, JQ1 causes MYC downregulation, which in turn leads to impairment of autophagy [15] and our microarray data from OCI-AML3 cells (unpublished) shows a significant increase in the mRNA of autophagy related genes LAMP2, SQSTM1, OPTN, and MAP1LC3B. However, in other AML cell lines with leukemia stem cell like properties (e.g., KG1 cells), JQ1 induced autophagy [16]. These cell lines, unlike the OCI-AML3 cells, are largely resistant to JQ1 induced apoptosis [17].

Clearly, the role of JQ1 in regulating autophagy is context-dependent, with the mutational status of the AML playing an important role. As mentioned above, this hypothesis is supported by our observations on patient samples. Moreover, Sakamaki et al., 2017 [18], show that bromodomain protein BRD4 is a repressor of autophagy in a panel of adherent cell lines, further demonstrating the contextual role of bromodomain proteins. In leukemia cell lines, it is possible that the expression of S100A8 and S100A9 determines the effect that bromodomain inhibition has on autophagy. High level S100A8 and S100A9 expression may predispose cells to JQ1 induced autophagy whereas cells with very low level S100A8 and S100A9 (e.g., KG1 cells) may respond to JQ1 by promoting autophagy.

Interestingly, we found that application of daunorubicin alone to AML cell lines results in a robust increase in S100A8 and S100A9 levels and that this increase could be overcome by JQ1. This suggests that daunorubicin induces chemoresistance by upregulating levels of these proteins via stimulation of autophagy. Likewise Yang et al., 2014, reported that doxorubicin and vincristine induced S100A8 expression in leukemia cell lines and that overexpression of S100A8 correlated with decreased sensitivity to these drugs. Daunorubicin has been shown to induce autophagy in leukemia cell lines by activation of the MEK/ERK pathway [19] which in turn induces expression of S100A8 and S100A9 in cancer cells [3].

Inhibition of S100A8 and S100A9 activity may have significant implications in cancer chemotherapy. Indeed inhibition of S100A9 by tasquinimod, a quinoiline-3-carboxamide derivative, through modulation of the tumour microenvironment has shown promising effects in preclinical models [20]. Further studies are required to determine the effects of JQ1 on autophagy in our experiments and to confirm that S100A8 and S100A9 are necessary and sufficient to overcome these effects. Targeting autophagy may prove to be a valuable tool in overcoming chemoresistance in some forms of AML [21].

## Figures and Tables

**Table 1:** Characteristics of primary AML samples.

**BSMS number**	**Sex**	**Age**	**Diagnosis**	**Cytogenetics**	**Gene Mutations**
0131	F	75	AML	Normal	None detected
1007	M	58	AML	Normal	NPM1 mutated
1010	M	76	AML	Normal	None detected
1013	M	76	AML	Normal	None detected
1015	M	85	AML	Normal	None detected
1019	M	33	AML	Inv16	None detected
1022	M	76	AML	Normal	None detected
1024	M	68	AML	Normal	None detected
1026	M	75	AML	Normal	None detected
1030	M	70	AML	Normal	DNMT3A, FLT3, TET, SRSF2 mutations
1031	M	60	AML	Normal	None detected
1032	M	52	AML	Normal	None detected
1033	M	72	AML	Normal	TET2, SRSF2, ASXL1, RUNX1 mutations
1036	M	74	AML	Normal	None detected
1037	M	48	AML	Normal	NPM1, FLT3 mutated
1038	M	74	AML	Monosomy 7	None detected
1041	F	81	AML	Normal	None detected
1045	M	57	AML	Normal	FLT3 mutated

## Data Availability

The data used to support the findings of this study are available from the corresponding author upon request.
